# *Candida glabrata *endophthalmitis following penetrating keratoplasty in a patient with negative donor rim culture

**DOI:** 10.1186/1471-2415-10-18

**Published:** 2010-06-11

**Authors:** Mohd Nor Muzaliha, Hussein Adil, Mohtar Ibrahim, Ismail Shatriah

**Affiliations:** 1 Department of Ophthalmology, School of Medical Sciences, Universiti Sains Malaysia, 16150 Kubang Kerian, Kelantan, Malaysia

## Abstract

**Background:**

*Candida glabrata *endophthalmitis following keratoplasty is rare and almost always associated with positive donor rim culture.

**Case presentation:**

A 63-year-old patient, diagnosed Fuch's endothelial dystrophy in both eyes underwent a penetrating keratoplasty in his right eye. He had multiple underlying medical problems, which included diabetes mellitus, hypertension, hypoadrenalism on oral dexamethasone and fatty liver secondary to hypertrigliseridemia. He developed multiple suture abscesses, corneal haziness, retrocorneal white plaques and a level of hypopyon two weeks after an uneventful penetrating keratoplasty in his right eye. Cultures of the donor button and the transport media culture were negative. *Candida glabrata *was isolated successfully from the aqueous and vitreous taps. He was treated with a combination of topical, intracameral, intravitreal and intravenous Amphotericin B. His final visual acuity remained poor due to the haziness of the corneal button.

**Conclusion:**

*Candida glabrata *endophthalmitis following penetrating keratoplasty can occur in negative donor rim and transport media cultures. The growth of the organism is facilitated by the patient's immunocompromised status. Awareness by the ophthalmologists and appropriate choice of antibiotics are mandatory in this challenging condition.

## Background

*Candida glabrata *is a very rare cause of endophthalmitis following penetrating keratoplasty. We reviewed 12 reported cases of C*andida glabrata *endophthalmitis following penetrating keratoplasty with varying onset from 1978 to 2010, in which most of them had positive donor rim culture.

We report on a case of *Candida glabrata *endophthalmitis following an uneventful penetrating keratoplasty in an immunocompromised patient. Cultures of the donor rim and transport media did not grow any microorganism. The organism was successfully isolated from both, the aqueous and vitreous taps. A prompt recognition and appropriate management are essential in this condition.

## Case presentation

A 63-year-old gentleman with Fuch's endothelial dystrophy in both eyes underwent a penetrating keratoplasty in his right eye. He had multiple underlying medical problems, which included diabetes mellitus, hypertension, fatty liver secondary to hypertrigliseridemia and hypoadrenalism. He had been on oral dexamethasone 5 mg morning and 2.5 mg night dose since 3 years ago.

The donor cornea was harvested eleven hours postmortem from a 56-year-old gentleman who died from peritonitis secondary to perforated appendicitis, with underlying pulmonary hypertension, valvular heart disease and chronic obstructive pulmonary disease. Donor tissue was stored for nine days at 4°C in Optisol-GS culture solution.

The pre-op visual acuity was 6/60 with no improvement with pinhole in the right eye and hand movement with good projection of light in the left eye. There was evidence of bullous keratopathy and guttata at the endothelial layer of cornea in both eyes. The right eye was pseudophakic. There was a dense cataract in his left eye. The intraocular pressure was normal in both eyes. Pre-operative B-scan ultrasonography confirmed an intact retina with clear vitreous. Systemic evaluations included full blood picture and chest x-ray did not reveal foci of infection.

The penetrating keratoplasty was uneventful. On day one postoperatively, his visual acuity was counting fingers at 2 feet. There was a generalized epithelial defect over the whole corneal button. The epithelium healed completely after ten days.

**Figure 1:**
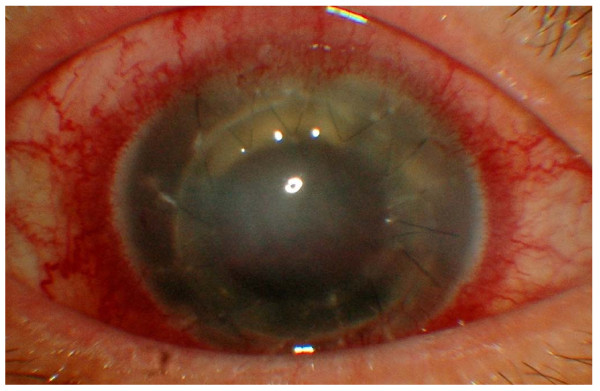
**Anterior segment photograph showing multiple suture abscesses and retrocorneal white plaques at the donor host interface at 2 weeks postoperative period**.

**Figure 2:**
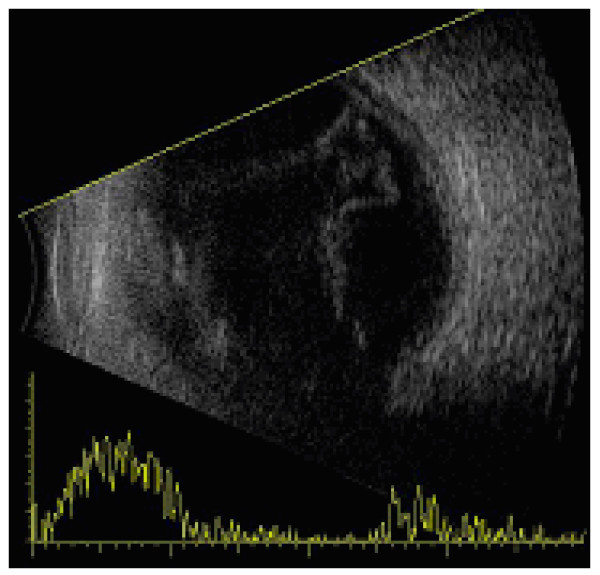
B-scan ultrasonography showing evidence of vitreous abscess with an intact retina in his right eye

At two weeks postoperative period, he complained of pain in the right eye and his visual acuity deteriorated to hand movement. The conjunctiva was congested and the corneal became hazy. There were multiple suture abscesses with retrocorneal plaques and a level of hypopyon (Figure 1). B-scan ultrasonography showed evidence of vitreous abscesses and a flat retina (Figure 2).

There was no organism isolated from either the cultures of donor corneal rim or transport medium. However, both the aqueous and vitreous taps grew *Candida glabrata*. Conjunctiva swab from the fellow eye and blood culture were negative.

He was treated with a combination of treatment, which included topical Amphotericin B 0.15% hourly and 0.5 mg/kg/day of intravenous Amphotericin B daily for the duration of six weeks. An intravitreal injection of 5 μg Amphotericin B was given once while performing the aqueous and vitreous taps. 5 μg of Amphotericin B was injected intracamerally two weeks later. He was monitored closely for signs of Amphotericin B toxicity.

He did not develop any toxic effect or serious complication during the therapy except for a minimal elevation of liver enzymes. He showed an encouraging clinical improvement, though his final visual acuity remained poor due to the haziness of the corneal button.

## Conclusions

*Candida glabrata *endophthalmitis following penetrating keratoplasty is very rare but has devastating effects. It typically occurs within the first and second week post penetrating keratoplasty [1-4]. In contrast, there were cases occurred as early as 10 hours post transplantation or as late as 5 months post surgery [5-7]. Our patient developed features of endophthalmitis at two weeks post penetrating keratoplasty.

*Candida glabrata*, formerly known as *Torulopsis glabrata *is a non-dimorphic/haploid yeast that has recently emerged as an important nosocomial pathogen [8]. It can be found in normal healthy skin, respiratory, genitourinary and gastrointestinal systems. It is a highly opportunistic pathogen of the urogenital tract and the blood stream.

The prevalence is high in HIV positive patients and the elderly, other risk factors include prolonged hospitalization and prior antibiotic use [8]. Infection with *Candida glabrata *can be mucosal or systemic, and occurs more commonly in an immunocompromised or a debilitated host.

Table 1 summarizes 12 reported cases of *Candida glabrata *endophthalmitis in the literature from 1998-2010. All of the cases had either positive cultures of donor rim or transport medium [1-7,9-12]. Both negative donor rim and transport medium cultures in postkeratoplasty endophthalmitis caused by *Candida glabrata *has never been reported previously. To the best of our knowledge, this is the first reported case of *Candida glabrata *postkeratoplasty endophthalmitis with negative donor rim and transport medium cultures.

**Table 1:** Comparison between previously reported cases of *Candida glabrata *endophthalmitis following penetrating keratoplasty

Author/Year	Age/Gender	Indication for PK	Onset of endophthalmitis	Culture	Treatment	Side effects
Larsen PA et al, 1978	76/Female	Aphakic bullous keratopathy	48 days	Positive (donor rim and fluid)	Topical and intravenous amphotericin B, oral and topical flucytosine	Renal toxicity; death
Cameron JA et al, 1991	22/Male	Keratoconus	7 days	Positive (donor rim)	Intravitreal amphotericin B, subconjunctival miconazole, topical natamycin,oral flucytocine	Not stated
Antonios et al, 1991	30/Male	Cornea scar	11 days	Positive (donor rim)	Not stated	Not stated
Cameron JA et al, 1998	46/Male	Cornea scar	3 weeks	Positive (donor rim)	Not stated	Not stated
Chapman FM et al, 1998	43/Male	Keratoconus	4 days	Positive (transport medium)	Topical,intracameral and subconjunctival amphotericin B	Nil
Garcia-Valenzuela E et al, 2005	85/Female	Fuch's endothelial dystrophy, pseudophakia	5 months	Positive (donor rim)	Oral fluconazole, intravitreal and intrastromal amphotericin B,	Not stated
Keyhani et al, 2005	82/Female	Pseudophakia corneal oedema	1^st ^to 2^nd ^week	Positive (donor rim)	Not stated	Not stated
Grueb M et al, 2006	26/Male	Keratoconus	10 hours	Positive (transport medium)	Systemic fluconazole, Topical and intracameral amphotericin B	Nil
Al Assiri A et al, 2006	69/Male	Trachomatous scarring	5 months	Positive (donor rim)	Topical and intracameral amphotericin B, systemic fluconazole	Not stated
Caldwell MC et al, 2009	57/Male	Post LASIK keratectasia	10 days	Positive (donor rim)	Oral fluconazole, intravitreal and topical amphotericin B, topical and oral voriconazole	Liver toxicity
Tappeiner C et al, 2009	70/Female	Fuch's endothelial dystrophy	1 day	Positive (donor rim)	Oral fluconazole, topical and subconjunctival amphotericin B, intravenous liposomal amphotericin B and intravenous caspofungin	Not stated
Tappeiner C et al, 2009	53/not stated	Fuch's endothelial dystrophy	1 day	Positive (donor rim)	Oral fluconazole and voriconazole; intracameral and subconjunctival Amphotericin B, intravenous liposomal amphotericin B and intravenous caspofungin	Renal toxicity
Current study	63/Male	Fuch's endothelial dystrophy	2 weeks	Negative (donor rim and transport medium) Positive (recipient AC and vitreous tap culture)	Topical, intravenous, intravitreal and intracameral amphotericin B	Nil

Cultures of the donor rim and transport medium were negative in our patient. However, the organism was isolated successfully from the aqueous and vitreous cultures. It seems most likely that an unrecognized *Candida glabrata *infection was present in the affected eye of the recipient. It is less likely that the contamination occurred during keratoplasty as it was performed under strictly sterile condition. We believe that the patient's immunocompromised status facilitated the infection process.

*Candida glabrata *is resistant to fluconazole and the other azoles group of antifungal but sensitive to Amphotericin B [8]. Several mechanisms of azole resistance have been identified, that include increased P-450-dependent ergosterol and an energy-dependent efflux pump of fluconazole, possibly via a multidrug resistance-type transporter [13,14]. Secondary in vitro resistance is the most common form of resistance in *candida glabrata *[15]. The reason for this rapid development of secondary antifungal resistance is unknown, but the haploid state of *candida glabrata *is thought to be a contributing factor [8].

In contrast, in vitro resistance of *candida glabrata *and *candida albicans *to ketoconazole and itraconazole accounts for only 15% but yet still significant [8]. Flucytosine resistance has been described extensively in *candida albicans *but not in *candida glabrata*. It has not been widely used in *candida glabrata *infections but may be useful in the future.

Fortunately, clinically significant amphotericin B resistance is still very uncommon among most *Candida *species and amphotericin B resistance has not been described in *candida glabrata*. In general, intracameral, intravitreal, intrastromal, subconjunctival, topical and systemic Amphotericin B have been used in the treatment of the *Candida glabrata *endophthalmitis [1,4-7,9,10,12].

Two patients developed renal toxicity after the treatment with intravenous Amphotericin, one of them subsequently died [10,12]. Liver toxicity had been reported in a patient who was treated concurrently with oral fluconazole [4]. Beside the above mentioned side effects, there were no documented serious complications in *Candida glabrata *endophthalmitis patients treated with various methods of Amphotericin B delivery (Table 1).

Fortunately, our patient did not develop any side effect during the course of Amphotericin B treatment. We had monitored him closely soon after commencing the treatment. We did not encounter retinal toxicity being reported as a side effect in our literature review though it has been mentioned in rabbit eyes [16,17].

Beside drugs resistance, relapse of *Candida glabrata *endophthalmitis is another challenging episode among the managing ophthalmologists. It had been reported months to years after primary eradication of the infection [4,6,18]. Thus, it is very essential to monitor closely these patients as the recurrence is common.

In conclusion, *Candida glabrata *endophthalmitis is uncommon but a potentially devastating complication following penetrating keratoplasty. A complete work-up is essential to identify the source of infection and prompt treatment is indicated. This case highlights that ophthalmologists should have a high index of suspicious especially when managing patients with immunocompromised status even with negative donor and transport media cultures.

## Consent

Written consent has been obtained from the patient for publication of this case report and accompanying images. A copy of written consent is available for review by the Editor-in-Chief of this journal.

## Competing interests

The authors declare that they have no competing interests.

## Authors' contributions

MMN participated in writing and editing the manuscript. AH examined and evaluated the patient. IM examined and evaluated the patient. SI participated in editing the manuscript. All authors read and approved the final manuscript.

## Pre-publication history

The pre-publication history for this paper can be accessed here:

http://www.biomedcentral.com/1471-2415/10/18/prepub
